# Assessment of Total Phenolic Content, In Vitro Antioxidant and Antibacterial Activity of *Ruta graveolens* L. Extracts Obtained by Choline Chloride Based Natural Deep Eutectic Solvents

**DOI:** 10.3390/plants8030069

**Published:** 2019-03-18

**Authors:** Valentina Pavić, Dora Flačer, Martina Jakovljević, Maja Molnar, Stela Jokić

**Affiliations:** 1 Department of Biology, Josip Juraj Strossmayer University of Osijek, Cara Hadrijana 8/A, 31000 Osijek, Croatia; dora.flacer@gmail.com; 2 Faculty of Food Technology Osijek, Josip Juraj Strossmayer University of Osijek, Franje Kuhača 20, 31000 Osijek, Croatia; mjakovljevic@ptfos.hr (M.J.); maja.molnar@ptfos.hr (M.M.); stela.jokic@ptfos.hr (S.J.)

**Keywords:** *Ruta graveolens*, natural deep eutectic solvents, extraction, antioxidant activity, antibacterial activity

## Abstract

Rue (*R. graveolens*) has been an extensively studied medicinal plant due to its rich phytochemicals content, such as furanocoumarins and flavonoids. The aim of this study was to determine the effects of varying extraction conditions on the total phenolic content, the antioxidant and antibacterial property of rue leaves crude extracts using deep eutectic solvents with different water content. These extraction conditions include the temperature and the extraction time. The extract obtained at 30 °C, with 20% water and at 90 min, with 13.3 µg mL^−1^ concentration, was found to possess the highest total phenolic content (38.24 ± 0.11 mg of GAE g^−1^ of DM) and the highest antioxidant activity (72.53 ± 0.31%). In this study, the same extract showed the best antibacterial efficiency against all the tested strains, especially gram-negative *P. aeruginosa*.

## 1. Introduction

Rue extracts, leaves and other parts of this plant (*R. graveolens* L.) have been used for a long time all over the world for different purposes. Within traditional medicine, rue was used as an antispasmodic, sedative and as a stimulant to start the menstrual cycle [1], while in some cultures, rue extracts were used as abortive agents [2], therefore it is not recommended for pregnant women [3]. Mediterranean traditional medicine utilized rue for treatments of various pulmonary conditions, swelling reduction and healing of wounds [4]. Its extracts can also be used as an antidote for snake and scorpion venoms [5]. A mixture of furoquinoline alkaloids is found in rue in concentrations of 1.5%, of which the significant are most arborin, arborinin and gamma-fagarin [6,7]. Acridone alkaloids (rutonium epoxide, hydroxylpudridone epoxide) are at their highest concentrations in the root [8], while other alkaloids include graveolin, graveolinin, kokusaginine, rutacridone and skimmianine [9]. Plants and their essential oils are rich in coumarin derivatives that contribute to their pharmacological activity. These derivatives include furocoumarines such as bergapten, psoralene, xanthoxanthine, xanthotoxin, isopimpinelin and rutamarin, and quinoline type alkaloids [10]. Rutin is a flavonoid found in many plants, including citrus fruits, and it has been shown that it can inhibit vascular endothelial growth factor in subtotic concentrations in vitro, and also acts as an angiogenesis inhibitor [11]. Rutin is considered to support and strengthen blood vessels, thus reducing blood pressure, and is used as an eye strengthener [12]. It is also a strong antioxidant, predominant in comparison with quercetin, acacetin, morphine, hispidulin, hesperidine and naringine [13]. Twelve phenolic compounds were found in various parts of the plant, including hydroxycinnamic acids and hydroxybenzoic acids. Phenolic acids and coumarins represent major groups of phenols in leaves and flowers, however, flavonoids were reported in low content (≤10% of total phenols) [14]. In more than 15 compounds found in the rue, in vitro antibacterial and antifungal activity was discovered [7]. Among them, acridone alkaloids are the most powerful antimicrobial compounds while coumarin inhibits bacterial and fungal growth only at high concentrations, while tested essential oil of rue did not show such activity. One of the studies suggests that *R. graveolens* extracts showed inhibitory activity against gram-positive bacteria such as *Staphylococcus aureus*, *Streptococcus pyogenes*, *Listeria monocytogenes* and *Bacillus subtilis* [15]. Other research has revealed that numerous rue components hinder DNA replication, thus inhibiting the reproduction of some viruses [16].

Natural deep eutectic solvents (NADES) are bio-based eutectic solvents consisting of two or more compounds which are overall primary plant-based metabolites such as organic acids, sugars, alcohols, amines and amino acids [17]. Deep eutectic solvents are often characterized as ”green“ solvents, since they are formed by mixing two, usually cheap, renewable and biodegradable components, forming a low melting point eutectic mixture [18,19]. Therefore, they are easy to prepare, with no need for further purification, chemically inert to water and have 100% atom economy during their synthesis [17,18,20]. Depending on the components mixed to form a DES, DES itself can extract polar or non-polar components depending on its structure [21,22]. Garcia et al. (2012) [21] have suggested that, considering their efficacy in polyphenol extraction, safety, sustainability and cost, DES can be a convenient alternative to methanol for this kind of extraction. 

The research conducted by Choi et al. in 2011 [23] showed that water could be incorporated in the solvent, and may not be evaporated. In our previous research [24], we performed an extraction of rutin from leaves of *Ruta graveolens* L. with different choline chloride based NADES in order to find an optimal extraction conditions regarding the best NADES; NADES to water ratio and extraction time. Also, when the optimal conditions regarding above mentioned were found, the further extraction optimization for chosen choline chloride-citric acid NADES regarding extraction temperature, time and water content was performed using response surface methodology (RSM) where it is evident that the rutin content remarkably increased with the increase of extraction temperature (from 30 °C to 70 °C). Furthermore, extraction time did not significantly affect the content of rutin, while higher water content showed slightly increased rutin content. In this work, we focused on the influence of various extraction conditions on the total phenolic content and the antioxidative and antibacterial activity of the crude extracts.

## 2. Materials and Methods

### 2.1. Chemicals

A rutin standard was purchased from the Sigma Aldrich (Taufkirchen, Germany). 2,2-Diphenyl-1-picrylhydrazyl (DPPH), triphenyl tetrazolium chloride (TTC), ascorbic acid (AA) and gallic acid were purchased from the Sigma Chemical Co. (St. Louis, MO, USA). Other solvents were obtained from J.T. Baker (Radnor, PA, USA). 

### 2.2. Plant Material

Dried leaves of *R. graveolens* L. were purchased from the Vextra d.o.o. herbal pharmacy (Mostar, Bosnia and Herzegovina) in the spring of 2016. The plant material was determined by Dragan Prlić, mag. biol. (Department of Biology, Josip Juraj Strossmayer University of Osijek, Croatia), and deposited a specimen in a herbarium at the Department of Biology, Josip Juraj Strossmayer University of Osijek, Croatia. Prior to extraction, the plant material was sifted through a laboratory mill. All measurements were performed in triplicate.

### 2.3. Preparation of NADESs

A choline chloride based deep eutectic solvent was prepared by mixing choline chloride and citric acid in a molar ratio of 2:1, as described in the previous literature [25]. The mixture was stirred and heated with constant stirring until a clear liquid is formed. Thereafter, the solvent was diluted with corresponding water content according to the Table 1 (10%, 20%, 27% and 30%), cooled down to room temperature and used for further extraction processess.

### 2.4. Extraction of Ruta graveolens L. with NADES and Experimental Design

50.0 mg of the ground rue leaves was was mixed with 1 mL of the selected solvent with stirring at a defined temperature for the selected time [24]. After extraction, a centrifugation was performed and supernatant decanted. The Box-Behnken Design (BBD), according to Bas and Boyacı (2007) [26], was chosen to create different extraction experiments and *Design-Expert^®^* commercial software (ver. 9, Stat-Ease Inc., Minneapolis, MN, USA) was used for data analysis. This optimization design allows us to find the most optimal levels of factors in the extraction process using NADES. The experimental design and proper statistical analysis with small number of runs in adjusting the NADES parameters became very popular in this field. Determination of rutin was performed in our prevoius work [24] by RP-HPLC method with UV detection on a Cosmosil 5C18-MS-II columns (Nacalai Tesque, Inc., Kyoto, Japan).

### 2.5. Determination of Total Phenolics Content

The total phenolics contents of NADES rue leaf extracts were determined by a spectrophotometric method which used Folin–Ciocalteu reagent. The standard calibration (0.018–0.30 mg mL^−1^) curve was plotted using gallic acid [27]. The results were derived from triplicate analyses, normalized against negative control of eutectic solvent with the corresponding water content according to the Table 1 and expressed as milligrams of gallic acid equivalents (GAE) per gram of dry mater (DM). 

### 2.6. 2,2-Diphenyl-1-Picrylhydrazyl (DPPH) Radical Scavenging Activity

Total antioxidant activities of NADES rue leaf extracts were determined using the DPPH radical scavenging assay described earlier [28]. 750 μL of the diluted plant extracts (final concentration 13.33 µg mL^−1^) was mixed with the same amount of 0.2 mM DPPH radical solution, so the final DPPH radical concentration was 0.1 mM. The mixture was well stirred and incubated at room temperature for 30 min. Ascorbic acid (AA) was used as a reference compound in concentration range 2–200 μg mL^−1^. All experiments were performed in triplicate. The absorbance decrease at 517 nm was measured, and DPPH scavenging activity was established using Equation (1):

DPPH activity = (A_b_ + A_s_) − A_m_)/A_b_ × 100 (1)

where A_b_ is the absorbance of 0.1 mM DPPH radical solution at λ = 517 nm, A_s_ is the absorbance of 0.1 mM extraction solution at λ = 517 nm, and A_m_ is the absorbance of 0.1 mM solution mixture of tested extracts and DPPH radical at 517 nm.

### 2.7. Antibacterial Susceptibility Testing

#### 2.7.1. Microorganisms and Growth Conditions

*Bacillus subtilis* and *Staphylococcus aureus* as two gram-positive, and *Escherichia coli* and *Pseudomonas aeruginosa* as gram-negative bacterial strains, were used to determine the antibacterial property of the NADES rue leaf extracts. These bacteria were isolates from various clinical specimens obtained from the Microbiology Service of the Public Health Institute of Osijek-Baranja County, Croatia. Working cultures were prepared from subcultures and grown overnight in Muller Hinton Broth (MHB) (Fluka, BioChemica, Germany) under optimal conditions (37 °C with 5% CO_2_ and 50% humidity). The antibacterial standard gentamicin (BioChemica, Germany) was dissolved in distilled water.

#### 2.7.2. Microorganisms and Growth Conditions

MIC values were determined by a modified microdilution method [29] as described in our previous work [30]. Assays were performed with sterile TPP 96-well plates (TPP Techno Plastic Products AG Trasadingen, Switzerland). A total of 100 μL of midlogarithmic-phase bacterial cultures (5 × 10^5^ CFU mL^−1^) in Mueller Hinton Broth were added to 100 μL of two-fold serially diluted extracts (250 to 0.122 μg mL^−1^). Wells containing bacterial inoculum without extracts (growth control) and wells containing only broth and solvent (background control) were included in each plate. The antibacterial standard gentamycin was co-assayed under the same conditions in concentration range 0.122–250 μg mL^−1^. After incubation at 37 °C for 24 h in an atmospheric incubator with 5% CO_2_ and 50% humidity, an additional incubation for three hours at 37 °C was performed with triphenyl tetrazolium chloride as a reducing agent indicator for microbial growth. The MIC value was defined as the lowest concentrations of extract at which there was no color change or visual turbidity due to microbial growth, derived from triplicate analyses, normalized against negative control of eutectic solvent with the corresponding water content according to the Table 1 and expressed as micrograms of gallic acid equivalents (GAE) per milliliter and also micrograms per milliliter. 

### 2.8. Statistical Data Processing

The comparison of extraction parameters with the total phenolics content and antioxidative and antibacterial activity was performed using the Pearson coefficient of correlation since data are continuous. Data obtained from this study were processed in the STATISTICA 12.0 statistical program (Statsoft, Inc., Tulsa, OK, USA). All tests were performed at a level of significance of α = 0.05.

## 3. Results and Discussion

In this work we performed 17 experiments changing process parameters for extraction of rue leaves with choline chloride: citric acid (2:1) DES. Our previous work showed that this DES is the most effective in rutin extraction, therefore we performed all other extractions in this solvent to investigate its effect on total phenol extraction, as well as antioxidative and antibacterial activity. A physical and chemical characterization of this solvent was determined by Popescu and Constantin (2014) [31]. A density of this DES was 1286.40–1320.80 kg m^-3^ in temperature range 393.15–354.15 K. The authors were not able to measure the viscosity of this solvent, due to its high viscosity. Experimental value for conductivity was 0.001 S/m. In this work, we focused on the total phenolics content, antioxidant and antibacterial activity of obtained NADES rue extracts in order to determine the optimal conditions for obtaining the highest total phenolics content, antioxidant and antibacterial activity. According to previous research, the extraction method and solvent greatly influence the amounts of antioxidant components in plant extracts [32]. 

NADES rue leaf extracts exhibited high total phenolics contents (TPC) ranging from 28.8 to 38.2 mg of GAE g^−1^ of DM (Table 1). The highest TPC (38. ± 0.11 mg of GAE g^−1^ of DM) was recorded in the rue extract obtained at 30 °C, 20% water and for 90 min (Run 10, Table 1), whereas the lowest content (28.84 ± 1.96 mg of GAE g^−1^ of DM) was found in the case of rue extract obtained at 30 °C, 20% water and for 30 min (Run 5, Table 1). Proestos et al. 2006 [33] found 4.3 ± 0.4 mg of GAE g^−1^ of DM in *R. graveolens* leaves dried with a freeze vacuum method. From the methanolic extracts of *Ruta graveolens* of south Indian origin by Benazir 2011 [34], 160 compounds was determined, representing terpenoids, aliphatic acids, flavonoids, alkaloids, quinones, alcohols, steroids and other compounds.

**Figure 1:**
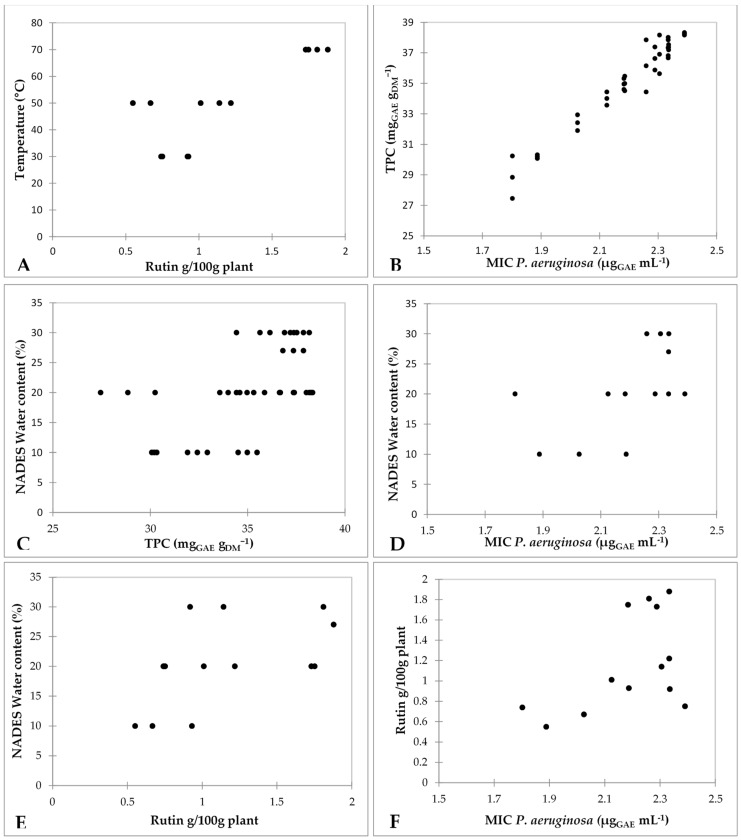
(**A**) Correlation between extraction temperature and rutin content of the NADES rue leaf extracts (r = 0.8229; *p* < 0.05). (**B**) Correlation between TPC and MIC *P. aeruginosa* of the NADES rue leaf extracts (r = 0.9733; *p* < 0.05). (**C**) Correlation between NADES water content and TPC of the NADES rue leaf extracts (r = 0.5439; *p* < 0.05). (**D**) Correlation between NADES water content and MIC *P. aeruginosa* of the NADES rue leaf extracts (r = 0.5588; *p* < 0.05). (**E**) Correlation between NADES water content and rutin content of the NADES rue leaf extracts (r = 0.5318; *p* < 0.05). (**F**) Correlation between rutin content and MIC *P. aeruginosa* of the NADES rue leaf extracts (r = 0.5061; *p* < 0.05).

The DPPH scavenging activities of the extracts (13.3 µg mL^−1^) are shown in Table 1. Rue extract obtained at 30 °C, 20 % water and for 90 min (Run 10, Table 1), also exhibited the highest antioxidant activity (72.53 ± 0.31%) amongst the investigated extracts, whereas the lowest antioxidant activity (57.54 ± 0.15%) was found in the case of rue extract obtained at previously determined [24] optimal conditions for rutin extraction 70 °C, 27% water and for 52 min (Run 17, Table 1). Pearson correlation analysis of all data points demonstrated significant (*p* < 0.050) moderate positive correlation between the rutin content and TPC results (*R* = 0.4926), and moderate positive correlation between the rutin content and extraction temperature (*R* = 0.8229), as shown in Figure 1.

NADES rue leaf extracts were tested for in vitro antibacterial activity against *E. coli*, *P. aeruginosa, B. subtilis*, and *S. aureus.* MIC values are shown in Table 2. 

As shown in Table 2, all tested extracts showed good antibacterial activities against *E. coli*, *P. aeruginosa*, *B. subtilis*, and *S. aureus*. The best antibacterial activity was seen against *P. aeruginosa*. This is in agreement with findings of Benazir 2011 [34], who found that the maximum zone of inhibition was noticed for *P. aeruginosa* in studies of methanolic extracts from *R. graveolens*. Among extracts, as shown in Table 2, the rue extract obtained at 30 °C, with 20% water and for 90 min (Run 5, Table 1) showed the lowest MIC 1.80 µg_GAE_ mL^−1^ (62.5 µg mL^−1^) against *P. aeruginosa*, and the highest MIC 4.7 µg_GAE_ mL^−1^ (125 µg mL^−1^) against *E. coli*, *B. subtilis*, and *S. aureus* was found in the case of rue extract obtained at previously determined [24] optimal conditions for rutin extraction 70 °C, 27% water and for 52 min (Run 17, Table 1). Same extract had the highest MIC among the extracts against *P. aeruginosa* (2.4 µg_GAE_ mL^−1^). Pearson correlation analysis of the MIC values expressed as µg mL^−1^ showed no correlation. Pearson correlation analysis of all data points between the MIC values expressed as µg_GAE_ mL^−1^ demonstrated a significant (*p* < 0.050) and very strong correlation between the MIC values of all tested bacteria and TPC results (*R* = 0.9733) and a moderate correlation between the eutectic solvent water content and MIC values (*R* = 0.5588), as shown in Figure 1. Extracts with higher TPC results showed higher MIC values. Specific rue leaf secondary metabolites like anthraquinone or saponins [34] have been suggested to have immediate antibacterial activity and recorded in *Aloe vera* [35]. The best antibacterial activity was found in extracts obtained at lower temperatures (30 °C), lower eutectic solvent water content (20%) and for longer extraction time (90 min). Anthraquinones stability is very susceptible to thermal decomposition, and it has been found that the determining factor for their thermal stability is their molecular structure [36]. The influence of temperature, pressure, and water flow rate on the extraction yield of anthraquinones from *Heterophyllaea pustulata* Hook f. aerial parts was determined by Barrera Vasquez et al. (2015) [37]. They point out that higher temperature caused lower yields of anthraquinones, which appears to be due to the thermal decomposition.

## 4. Conclusions

In this work, the influence of various extraction conditions of the extraction of rue leaves using NADES on the total phenolic content and the antioxidative and antibacterial activity against gram-positive and gram-negative human pathogens was performed. The highest total phenolic content (38.24 ± 0.11 mg of GAE g^−1^ of DM) and highest antioxidant activity (72.53 ± 0.31%) at 13.3 µg mL^−1^ concentration between the investigated NADES rue leaf extracts was achieved in the extract obtained at 30 °C, with 20% water and for 90 min. In this study, the same extract showed the best antibacterial efficiency against all the tested strains, especially gram-negative *P. aeruginosa*. According to the results acquired in the present work, the rutin content of the NADES rue leaf extracts seems to not affect the antibacterial activity of the crude extracts. Extracts with higher TPC results showed higher MIC values. In next study, it may be necessary to determine the presence of many secondary metabolites in obtained NADES rue leaf extracts in order to reveal antibacterial compounds.

## Figures and Tables

**Table 1:** Experimental matrix and Total phenolics content (TPC) in NADES rue leaf extracts expressed as mg of GAE g^−1^ of DM and 2,2-diphenyl-1-picrylhydrazyl (DPPH) radical scavenging activity expressed as % DPPH radical scavenging activity at 13.3 µg mL^−1^ concentration.

Run	NADES Water Content (%) *	Time (min) *	Temp. (°C) *	Rutin g/100g Plant *	TPC (mg_GAE_ g_DM_^−1^)	DPPH Radical Scavenging Activity (%)
1	10	30	50	0.55	30.19 ± 0.16	67.59 ± 0.98
2	20	60	50	1.01	34.02 ± 0.61	65.79 ± 0.15
3	20	30	70	1.75	34.95 ± 0.51	65.61 ± 0.15
4	20	60	50	1.19	ND	ND
5	20	30	30	0.74	28.84 ± 1.96	66.25 ± 0.15
6	10	60	30	0.93	34.99 ± 0.68	57.63 ± 0.363
7	20	60	50	1.08	ND	ND
8	30	60	30	0.92	37.36 ± 0.24	70.91 ± 0.31
9	30	60	70	1.81	36.14 ± 2.41	68.04 ± 0.47
10	20	90	30	0.75	38.24 ± 0.11	72.53 ± 0.31
11	20	60	50	1.22	37.33 ± 0.95	70.02 ± 0.26
12	10	90	50	0.67	32.42 ± 0.73	62.29 ± 0.05
13	20	90	70	1.73	36.62 ± 1.06	68.31 ± 0.11
14	30	90	50	1.67	ND	ND
15	10	60	70	1.07	ND	ND
16	30	30	50	1.14	36.89 ± 1.79	64.09 ± 0.15
17	27	52	70	1.88	37.33 ± 0.72	57.54 ± 0.15

* The data are from Molnar et al. (2018) [24]. ND: not determined. Data expressed as mean ± S.D.

**Table 2:** Minimum inhibitory concentrations (MIC) of NADES rue leaf extracts against *Escherichia coli*, *Pseudomonas aeruginosa, Bacillus subtilis*, and *Staphylococcus aureus* (μg_GAE_ mL^−1^ and μg mL^−1^).

Run	Minimum Inhibitory Concentration							
	*E. coli*		*P. aeruginosa*		*B. subtilis*		*S. aureus*	
	μg_GAE_ mL^−1^	μg mL^−1^	μg_GAE_ mL^−1^	μg mL^−1^	μg_GAE_ mL^−1^	μg mL^−1^	μg_GAE_ mL^−1^	μg mL^−1^
1	3.77	125	1.89	62.5	3.77	125	3.77	125
2	4.25	125	2.13	62.5	4.25	125	4.25	125
3	4.37	125	2.18	62.5	4.37	125	4.37	125
4	ND	ND	ND	ND	ND	ND	ND	ND
5	3.61	125	1.80	62.5	3.61	125	3.61	125
6	4.37	125	2.19	62.5	4.37	125	4.37	125
7	ND	ND	ND	ND	ND	ND	ND	ND
8	4.67	125	2.34	62.5	4.67	125	4.67	125
9	4.52	125	2.26	62.5	4.52	125	4.52	125
10	4.78	125	2.39	62.5	4.78	125	4.78	125
11	4.67	125	2.33	62.5	4.67	125	4.67	125
12	4.05	125	2.03	62.5	4.05	125	4.05	125
13	4.58	125	2.29	62.5	4.58	125	4.58	125
14	ND	ND	ND	ND	ND	ND	ND	ND
15	ND	ND	ND	ND	ND	ND	ND	ND
16	4.61	125	2.31	62.5	4.61	125	4.61	125
17	4.67	125	2.33	62.5	4.67	125	4.67	125
G	ND	0.976	ND	0.976	ND	1.95	ND	3.91

ND: not determined. G-gentamicin.
